# Youthful Processing Speed in Older Adults: Genetic, Biological, and Behavioral Predictors of Cognitive Processing Speed Trajectories in Aging

**DOI:** 10.3389/fnagi.2017.00055

**Published:** 2017-03-10

**Authors:** Nicholas T. Bott, Brianne M. Bettcher, Jennifer S. Yokoyama, Darvis T. Frazier, Matthew Wynn, Anna Karydas, Kristine Yaffe, Joel H. Kramer

**Affiliations:** ^1^School of Medicine, Stanford University, StanfordCA, USA; ^2^Neurology, Memory and Aging Center, University of California San Francisco, San FranciscoCA, USA; ^3^Neurosurgery and Neurology, School of Medicine, University of Colorado, AuroraCO, USA; ^4^Psychiatry, University of California, San Francisco, San FranciscoCA, USA

**Keywords:** processing speed, cognitive aging predictors, APOE E4 allele, white matter, IL-6, insulin, physical exercise

## Abstract

**Objective:** To examine the impact of genetic, inflammatory, cardiovascular, lifestyle, and neuroanatomical factors on cognitive processing speed (CPS) change over time in functionally intact older adults.

**Methods:** This observational study conducted over two time points, included 120 community dwelling cognitively normal older adults between the ages of 60 and 80 from the University of California San Francisco Memory and Aging Center. Participants were followed with composite measures of CPS, calculated based on norms for 20–30 year-olds. Variables of interest were AD risk genes (APOE, CR1), markers of inflammation (interleukin 6) and cardiovascular health (BMI, LDL, HDL, mean arterial pressure, fasting insulin), self-reported physical activity, and corpus callosum (CC) volumes. The sample was divided into three groups: 17 “resilient-agers” with fast and stable processing speed; 56 “average-agers” with average and stable processing speed; and 47 “sub-agers” with average baseline speed who were slower at follow-up.

**Results:** Resilient-agers had larger baseline CC volumes than sub-agers (*p* < 0.05). Resilient-agers displayed lower levels of interleukin-6 (IL-6) and insulin (*p*s < 0.05) than sub-agers, and reported more physical activity than both average- and sub-agers (*p*s < 0.01). In a multinomial logistic regression, physical activity and IL-6 predicted average- and sub-ager groups. Resilient-agers displayed a higher frequency of APOE e4 and CR1 AA/AG alleles.

**Conclusion:** Robust and stable CPS is associated with larger baseline CC volumes, lower levels of inflammation and insulin, and greater self-reported physical activity. These findings highlight the relevance of neuroanatomical, biological, and lifestyle factors in the identification and prediction of heterogeneous cognitive aging change over time.

## Introduction

Cognitive processing speed (CPS) reaches its zenith during early adulthood (Salthouse, 1996) and declines from midlife onward (Salthouse, 2009), with age-related cognitive slowing widely reported (Cerella and Hale, 1994; Jenkins et al., 2000). Moreover, CPS is a uniquely strong predictor of age-related cognitive declines among older adults, including those needing help with activities of daily living (Salthouse and Ferrer-Caja, 2003; Wahl et al., 2010). Nevertheless, there is considerable variability in the degree to which cognitive functioning changes with age, and recent studies have identified a small number of individuals, who appear resilient against such age-related cognitive decline within the specific domain of episodic memory (Rogalski et al., 2013; Gefen et al., 2014, 2015; Sun et al., 2016). Whether similar resilience exists within different cognitive domains is unknown. This study sought to identify and characterize individuals resilient to age-related slowing in CPS over time.

Biological aging models posit upstream causal factors such as lifestyle variables and genetics interacting with inflammatory status and vascular health to affect downstream factors such as neurodegeneration and cognitive function (DeCarlo et al., 2014). Within such a model, the cause of age-related cognitive slowing is likely a complex cascade of processes. Declines in CPS have been associated with genetic risk factors for Alzheimer’s disease (AD) (Chibnik et al., 2011; Batterham et al., 2013), reduction of white matter volume and integrity (Jokinen et al., 2007, 2012; Kerchner et al., 2012), vascular health (Crichton et al., 2012; Jacobs et al., 2013), chronic levels of systemic inflammation (Bettcher et al., 2014; Palta et al., 2015), and levels of physical activity (Lo et al., 2014; Nouchi et al., 2014).

Less well-understood is which factors drive changes in CPS over time. We reasoned that studying the extremes of CPS change over time provides an opportunity to understand the unique health profile (genetic, biological, and behavioral factors) related to its preservation and decline, some of which may be amenable to change through lifestyle choices (Blazer et al., 2015). We hypothesized that “resilient-agers” with unusually robust and stable CPS over time would be associated with fewer genetic AD risks, greater white matter health, lower levels of inflammation, fewer cardiovascular risk factors, and increased rates of physical activity.

## Materials and Methods

### Subjects

This was an exploratory observational study conducted over two time points. We started with a convenience sample of 165 neurologically and functionally intact older adults (aged 60 years and older) from existing research cohorts at the UCSF Memory and Aging Center based on the availability of composite CPS data. Each potential subject underwent a complete history, neurological examination, a functional assessment, and an hour-long neuropsychological screening battery, followed by a consensus conference for the determination of diagnosis and suitability for the study. Subject inclusion was based upon a Mini-Mental State Exam score of >25 (Folstein et al., 1975), a Clinical Dementia Rating score of zero, no symptoms of cognitive impairment during the previous year as endorsed by either the patient or informant, and no findings during examination that raised concern for emerging cognitive decline. Exclusion criteria included any contraindication to magnetic resonance imaging (MRI), major psychiatric disorder, neurological conditions affecting cognition (e.g., Parkinson’s disease, epilepsy), head injury with loss of consciousness of 30-min or more, diagnosis of MCI or dementia, history of brain tumor, sensory or motor disability, active substance abuse, significant systemic medical illnesses, current medications likely to affect CNS functions, current depression (Geriatric Depression Scale Score greater than 15 of 30), or insulin-dependent diabetes. Information regarding medical history and medication use was secured via participant self-report. All subjects continued to meet both inclusion and exclusion criteria throughout the duration of the study period. The UCSF committee on human research approved the study, and all subjects provided written, IRB-approved informed consent before participating.

### Cognitive Processing Speed Testing

Each participant was administered a fixed battery of seven visuospatial tasks, with tasks including between 1 and 4 conditions for a total of 14 task conditions (see Supplementary Data Sheet 1). Administration time for the complete battery was approximately 25 min. A scaled response latency z-score for each task condition was calculated and compared to those among a sample of young adult controls (*n* = 40; 16 males; age 24 ± 3.1 years; education 16 ± 1.6 years). The average of the 14 z-scores for each older subject yielded a composite z-score, with higher scores denoting slower speed relative to the young adult controls. This comparison of mean reaction times between older subjects and younger controls has previously been validated (Kerchner et al., 2012).

All task conditions were programmed on a PC using Eprime software (Psychology Software Tools, Inc., Sharpsburg, PA, USA^1^). Experiments were administered on a standard 15.4″ Dell Latitude D830 laptop. Subjects were presented with stimuli on the display and instructed to make visuospatial judgments. Standard instructions were for subjects to respond as quickly and accurately as possible. All tasks required a binary decision (yes/no or left/right), and subjects were asked to use the index and middle finger of their right hand to press the key on a keyboard corresponding to their answer choice. Tasks commenced with between 5 and 10 practice trials; if accuracy on these trials fell below 70%, an additional round of practice trials was administered before proceeding. The first two experimental trials in each task condition served as ‘cushion’ trials and were not included in the analyses. The median response latency of the subsequent 20 experimental trials of each task condition was recorded for analysis.

### Processing Speed Change

**FIGURE 1:**
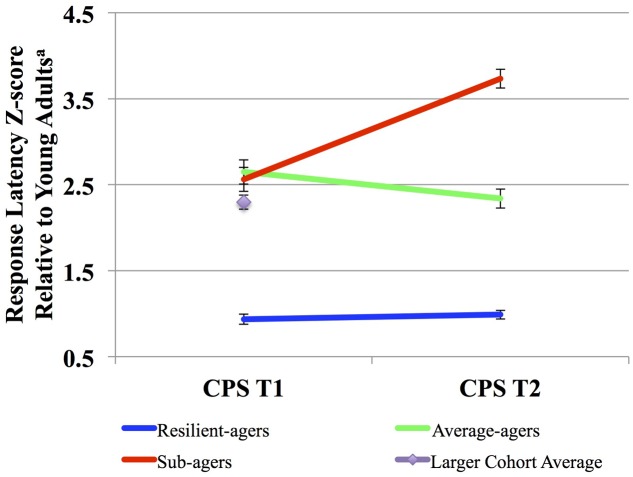
**Cognitive processing speed (CPS) trajectories.** ^a^Scaled response latency composite z-score compared to those among a sample of young adult controls (*n* = 40; age: 24 ± 3.1 years). Higher scores denote slower speed relative to the young adult controls.

We defined three CPS thresholds of change over two time points in order to distinguish both fast and stable CPS, shown below in **Figure 1**. The battery of CPS tasks has been described previously (see Supplemental Material) (Kerchner et al., 2012). Resilient-agers included those individuals whose baseline CPS performance was within 1.25 standard deviations (SD) of the young adult comparison group. Baseline CPS performance within 1.25 SD was the threshold for an above average performance compared to the larger older adult cohort (>75%). Additionally, resilient-agers changed no more than 0.5 SD at follow-up. A 0.5 SD cut-point in the observed distribution has been employed as the minimally clinically important change over time from baseline (Small et al., 2012; Brouillette et al., 2016). A 0.5 SD change in CPS z-score corresponded to a 0.5 SD change from baseline in the larger older adult cohort. Average-agers were slower than the young controls at baseline (>1.25 SD) but also changed no more than 0.5 SD at follow-up. Sub-agers were slower than the young controls at baseline (>1.25 SD) and continued to decline (>0.5 SD) at follow-up. From this sample we identified 120 subjects fitting our *a priori* baseline and follow-up CPS parameters for resilient-agers, average-agers and sub-agers. These criteria resulted in 17 resilient-agers, 56 average-agers, and 47 sub-agers. Mean length of follow-up was 2.5 (0.80) years. Baseline CPS of average-agers and sub-agers was comparable to the larger cohort of older adults from which they were drawn (*n* = 283; z-score = 2.3 ± 1.4). Demographic variables are summarized in **Table 1**.

**Table 1:** Demographic, clinical, and health profile variables by CPS trajectory and effect size comparisons.

	Resilient-agers^a^	Average-agers^a^	Sub-agers^a^	Resilient vs. Average^b^	Resilient vs. Subs^b^	Average vs. Subs^b^
**Demographic/clinical characteristics**					
No.	17	56	47			
Age, y	69.2 (0.96) (62–77)	70.9 (0.65) (63–79)	72.0 (0.68) (62–79)	-0.38	-0.64	-0.23
Sex, % F	42	51	47			
Education, y	18.4 (0.51)	17.7 (0.28)	17.4 (0.35)	0.33	0.44	0.13
GDS (total)^e^	2.6 (0.85)	2.5 (0.37)	3.1 (0.50)	0.03	-0.15	-0.20
MMSE (total)^f^	29.3 (0.24)	29.2 (0.11)	29.3 (0.15)	0.11	0.00	-0.11
CPS z-scores						
Time 1	0.94 (0.06)	2.6 (0.14)	2.6 (0.18)			
Time 2	0.99 (0.05)	2.3 (0.11)	3.7 (0.22)			
Follow-up (years)	2.24 (0.22)	2.57 (0.11)	2.49 (0.11)	-0.38	-0.30	0.10
**Health profile variables**					
*APOE* e4 allele	59% (10)	32% (18)	23% (11)	0.61	**0.85^d^**	0.24
*CR1* AA/AG allele	65% (11)	48% (25)	30% (14)	0.38	**0.81^c^**	0.43
CC volume^g^	3101.3 (93.7)	2879.6 (51.2)	2803.1 (54.8)	0.58	**0.78^c^**	0.20
IL-6, pg/ml^h^	0.7 (0.21)	1.4 (0.22)	2.1 (0.35)	-0.64	-**0.94^c^**	-0.41
BMI^i^	23.8 (0.54)	25.0 (0.39)	25.9 (0.65)	-0.48	-0.63	-0.25
Insulin (uU/ml)^j^	6.6 (0.83)	9.2 (0.89)	12.2 (1.4)	-0.57	-**0.84^c^**	-0.40
HDL-C (mg/dL)^j^	69.8 (4.1)	70.9 (3.3)	62.4 (4.0)	-0.06	0.36	0.36
LDL-C (mg/dL)^j^	130.3 (9.8)	127.8 (6.3)	117.5 (5.6)	0.07	0.37	0.27
MAP^k^	146.6 (56.9)	149.3 (27.8)	152.5 (33.4)	-0.01	-0.03	-0.01
PASE^l^	194.3 (23.5)	128.2 (9.5)	132.7 (11.1)	**0.99^d^**	**0.87^d^**	-0.07


*^a^Values are mean (SE); % (n); (range). Higher CPS z-scores represent worse performance. ^b^Values are Cohen d effect size. ^c^Significant at threshold, *p* < 0.05. ^d^Significant at threshold, *p* < 0.01. ^e^Geriatric Depression Scale (GDS) scores range from 0 to 15 with higher scores indicating a greater number of depressive symptoms. ^f^Mini-mental Status Examination (MMSE) scores range from 0 to 30 with lower scores indicating more significant cognitive deficits. ^g^Volumetric data were available in a subsample of 16 Resilient-agers, 54 Average-agers, and 47 Sub-agers. ^h^Interleukin-6 (IL-6) data were available in a subsample of 10 Resilient-agers, 39 Average-agers, and 34 Sub-agers. ^i^Body mass index (BMI) data were available in a subsample of 16 Resilient-agers, 52 Average-agers, and 42 Sub-agers. ^j^Insulin and cholesterol data were available in a subsample of 12 Resilient-agers, 43 Average-agers, and 39 Sub-agers. ^k^Mean arterial pressure (MAP) data were available in a subsample of 16 Resilient-agers, 56 Average-agers, and 46 Sub-agers. ^l^Physical Activity Scale for the Elderly (PASE) data were available in a subsample of 10 Resilient-agers, 37 Average-agers, and 30 Sub-agers. PASE scores range from 0 to 361 with higher scores indicating greater amounts of physical activity.*

### Variables of Interest

Variables of interest representing both upstream and downstream biological aging factors included genetics, white matter volume, inflammatory markers, vascular risk factors, and lifestyle factors that have been found to play a role in the slowing of CPS among older adults. For genetics, we selected two well-established AD risk genes. *APOE* was investigated due to the role of the e4 allele as a risk factor for AD, and its association in the slowing of CPS (Bartzokis et al., 2007). *CR1* was also investigated given the A allele’s role as a risk factor for late-onset AD, and its role in the acceleration of cognitive decline, including declines in CPS (Lambert et al., 2009; Chibnik et al., 2011; Sweet et al., 2012). Frequency of *APOE* and *CR1* genotypes were compared across groups. Our marker of white matter health was calculation of total corpus callosum (CC) volume (sum of posterior, mid-posterior, central, mid-anterior, and anterior regional volumes), which has been associated with CPS in older adults (Jokinen et al., 2007, 2012). The CC is the most important commissural tract in the brain containing myelinated axons transversing the subcortical white matter that mediate the transfer and integration of sensory, cognitive, and motor information between hemispheres. Studies have demonstrated the relationship between CC volume and myelin integrity (Hugenschmidt et al., 2008), and the relationship of each to CPS (Fling et al., 2011; Kerchner et al., 2012). Our primary marker of inflammation was calculation of peripheral level of interleukin-6 (IL-6), which has been associated with declines in CPS (Bettcher et al., 2014; Palta et al., 2015). Vascular risk factors of interest demonstrating relationships with CPS include measurement of body mass index (BMI), calculated as [weight (kg)/height (m)2], low-density lipoprotein (LDL), high-density lipoprotein (HDL), mean arterial pressure (MAP), calculated as [(2 × diastolic)+systolic], and levels of fasting insulin (Yaffe et al., 2004; Jefferson et al., 2015). Physical activity was calculated by self-report using the Physical Activity Scale for the Elderly (PASE), which asks subjects to recall their level of physical activity over the previous 1 week (Forsen et al., 2010).

### Laboratory Measures

Fasting blood samples were collected for estimation of HDL, LDL, insulin, and genotyping. Plasma and serum separator tubes were used to collect blood specimens. Tubes were left to clot at room temperature for 30–60 min and placed into EDTA plasma tubes. The blood was then centrifuged at 2500 rpm at room temperature for 15 min. Plasma and serum were stored at -80°C until samples were analyzed. LDL, HDL, fasting glucose and insulin samples were assessed in the UC Davis Medical Center Clinical Laboratory. After fasting glucose and insulin samples were collected, HOMA-IR ratio was computed as [(fasting insulin (μU/mL) × fasting glucose (mg/dL))/405]. IL-6 was measured using a Quantikine ELISA kit from R&D systems (Minneapolis, MN, USA) in the laboratory of Dr. Ralph Green at the UC Davis Medical Center (Sacramento, CA, USA).

### Genotyping

Genomic DNA was extracted from peripheral blood using standard protocols (Gentra PureGene Blood Kit, Qiagen, Inc., Valencia, CA, USA). Genotyping was performed using either TaqMan or Sequenom genotyping. TaqMan Allelic Discrimination Assay was used for *APOE* genotyping (rs429358 and rs7412), and was conducted on an ABI 7900HT Fast Real-Time PCR system (Applied Biosystems, Foster City, CA, USA) according to manufacturer’s instructions. Sequenom iPLEX Technology (Sequenom, San Diego, CA, USA) was used for *CR1* genotyping (rs6656401). The SpectroAquire and MassARRAY Typer Software packages (Sequenom, San Diego, CA, USA) were used for interpretation and Typer analyzer (v3.4.0.18) was used to review and analyze data.

### Neuroimaging

Magnetic resonance imaging scanning was performed on a 3T Siemens scanner (Siemens, Iselin, NJ, USA), using a protocol that included a T1-weighted 3D MPRAGE sequence (TR/TE/TI 2300/3/900 ms; flip angle 9u; sagittal acquisition with FOV 2566240 mm^2^ and 1 mm thick slices; matrix 2566240 with 160 slices yielding 1 mm^3^ isotropic voxels). Structural MR images were analyzed using the FreeSurfer image analysis suite^2^. After initial automated segmentation through FreeSurfer version 5.1, each subject’s image was individually checked for quality and accuracy of segmentation. Remaining inaccuracies in either gray and white matter segmentation or pial boundaries and residual skull fragments were manually corrected with FreeSurfer’s built-in editing software. These cases were reprocessed and final volumes were recalculated. Intracranial volume (ICV) was calculated based on FreeSurfer’s own eTIV (estimated total ICV) metric, which uses atlas normalization as well as the relationship between the linear transform to MNI305 space and ICV. Region of interest was the CC (Fischl et al., 2002).

### Data Analysis

Statistical analyses were performed using PASW 21.0 for Mac (SPSS, Inc., Chicago, IL, USA). Demographic variables were compared using analysis of variance with chi square for dichotomous variables and Tukey *post hoc* tests and α = 0.05. General linear models were performed to determine group differences in variable scores of interest. Pairwise comparisons with Tukey *post hoc* analysis were used to evaluate differences among adjusted means. Bonferroni correction was used to evaluate differences among adjusted means for genotyping and neuroanatomical models. *p-*values < 0.05 for these comparisons were considered significant. The number of genetic and vascular measures investigated was not corrected for multiple comparisons. Given the exploratory nature of this study, and the limited corpus of literature on this topic, we chose to risk an increase in type I errors over type II errors. A multinomial logistic regression with CPS groups as the dependent variable and significant clinical variables as the predictors was performed to determine which clinical variables, if any, predicted CPS groups.

## Results

**FIGURE 2:**
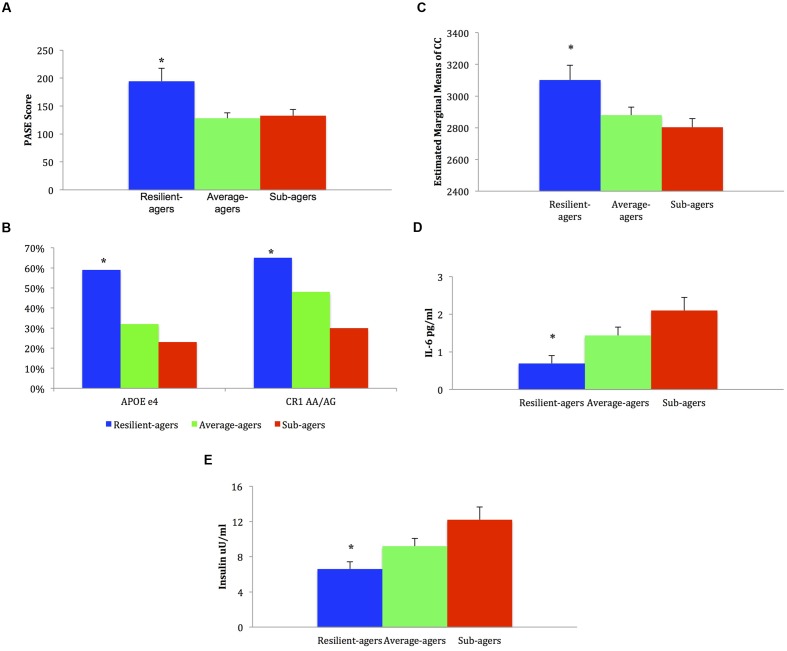
**Mean, standard error, and frequencies of health profile variable scores.** **(A)** Baseline physical activity: ^∗^ resilient-agers > average-agers and sub-agers. **(B)** Genotype frequency %: ^∗^ resilient-agers > sub-agers. **(C)** Baseline corpus callosum volume: ^∗^ resilient-agers > sub-agers. **(D)** Baseline levels of IL-6: ^∗^ resilient-agers < sub-agers. **(E)** Baseline levels of insulin: ^∗^ resilient-agers < sub-agers.

There were no group differences in age, sex, or education. The raw mean scores, SD, and effect sizes of test performance by group for all demographic, clinical, and health profile variables are presented in **Table 1**. Graphical representation of significant health profile variables is presented in **Figure 2**.

### APOE and CR1 Genotyping

Analysis of *APOE* e4 allelic frequency revealed that of the 17 resilient-agers, 10 (59%) had an e4 allele. In contrast, only 18 average-agers (32%) and 11 sub-agers (23%) had an e4 allele. Chi square analysis of *APOE* e4 allele frequency was significant (χ^2^ = 7.15; *p* = 0.028) with greater frequency of an e4 allele in the resilient-agers than sub-agers. Analysis of *CR1* rs6656401 AA/AG allelic frequency revealed that of the 17 resilient-agers, 11 (65%) had AA/AG alleles, versus 6 (35%) with the GG allele. In contrast, only 25 average-agers (48%) and 14 sub-agers (30%) had AA/AG alleles. Chi square analysis of AA/AG allelic frequency was significant (χ^2^ = 7.16; *p* = 0.028) with greater frequency of AA/AG alleles in the resilient-agers than sub-agers. Descriptive analysis of the 17 resilient-agers revealed that 7 (41%) were carriers of both *CR1* AA/AG alleles and the *APOE* e4 allele.

### Baseline Corpus Callosum Volume

Resilient-agers had larger CC volume at baseline than sub-agers (*p* < 0.05). There was no difference in CC volume between resilient-agers and average-agers.

### Baseline Level of Peripheral Inflammation

Resilient-agers exhibited lower levels of IL-6 at baseline than sub-agers (*p* < 0.05). There was no difference in IL-6 levels between resilient-agers and average-agers.

### Baseline Cardiovascular Risk Measures

No differences between groups were observed on baseline levels of BMI, HDL, LDL, or MAP. Resilient-agers exhibited lower levels of fasting insulin than sub-agers (*p* < 0.05). There was no difference in insulin level between resilient-agers and average-agers.

### Baseline Physical Activity Level

Resilient-agers reported more activity than both average- and sub-agers, endorsing more physical activity on the PASE at baseline (*p* < 0.01).

### Secondary Analysis

To further explore the contributions of these factors we constructed a multinomial logistic forced entry regression with the three CPS groups as the dependent variable and all significant continuous outcome variables as predictors (PASE, insulin, IL-6, and CC volume). We also included age as a covariate. Due to availability of data for the significant outcome variables, the sample size for this model included seven resilient-agers, 27 average-agers, 22 sub-agers. The multinomial logistic regression indicated that the overall model was significant (*p* = 0.024; Nagelkerke pseudo-R^2^ = 0.358). Likelihood ratio tests indicated that IL-6 [χ^2^(2,56) = 7.20; *p* = 0.027)] and PASE [χ^2^(2,56) = 6.16; *p* = 0.046)] were significant predictors. Parameter estimates with resilient-agers as the reference group indicated PASE as a significant predictor with respect to average-agers [χ^2^(1,27) = 4.60; unstandardized beta = -0.019; 95% CI = 0.963 to 0.968; *p* = 0.032]. Parameter estimates with resilient-agers as the reference group indicated PASE [χ^2^(1,22) = 4.20; unstandardized beta = -0.019; 95% CI = 0.963 to 0.999; *p* = 0.040] and IL-6 [χ^2^(1,22) = 3.90; unstandardized beta = 1.64; 95% CI = 1.011 to 26.28; *p* = 0.048] as significant predictors with respect to sub-agers.

## Discussion

In the present exploratory study we defined and identified three CPS change thresholds in older adults based upon baseline and 2.5-year follow-up CPS performance. The major findings of this paper are that when compared with average- and sub-agers, resilient-agers endorsed more physical activity at baseline, and exhibited lower levels of IL-6 and fasting insulin, and larger total CC volume than sub-agers at baseline. Contrary to expectations, we observed a higher frequency of two AD risk alleles, *APOE* e4 and *CR1* AA/AG, in the resilient-agers than in the sub-agers.

Our findings are consistent with reports highlighting the role of chronic low-grade inflammation in cognitive aging processes (Bettcher and Kramer, 2013; Franceschi and Campisi, 2014). Specifically, circulating levels of IL-6 increase with age (Albani et al., 2009) and have been associated with rate of cognitive decline in older adults (Yaffe et al., 2004; Dik et al., 2005; Mooijaart et al., 2013). An inverse relationship between IL-6 and CC fractional anisotropy has been reported, the effect of which increased with age in typically aging older adults. This cross-sectional study also reported that higher levels of IL-6 were related to slower processing speed (Bettcher et al., 2014). This is the first study to demonstrate the role of IL-6 on speed of cognitive processing over time in a sample of older adults. Moreover, the results of the multinomial regression suggest that IL-6 may play a particularly significant role in the maintenance of CPS.

The role of vascular factors in driving cognitive decline in healthy and pathological populations has a growing body of support (Crichton et al., 2012). Insulin resistance (fasting insulin and HOMA-IR) has previously been associated with declines in cognition in middle-aged and older adults, including slower CPS (Laakso, 1993; Young et al., 2006; Tan et al., 2011; Frazier et al., 2015). It is reasonable to hypothesize that insulin resistance may contribute to slower processing speed via changes in white matter integrity. A recent study reported that greater insulin resistance as measured by HOMA-IR levels was associated with alterations on diffusion tensor imaging (DTI) in the CC and connection tracts in frontal white matter (Ryu et al., 2014). Although we cannot determine the causal nature of this association, this study extends the relationship between measures of insulin resistance and CPS to include typically aging older adults.

The relationships between preserved volume and white matter integrity of the CC and CPS have also demonstrated robust positive associations (Jokinen et al., 2007, 2012; Kerchner et al., 2012). Overall CC atrophy is associated with slower CPS in older adults with age-related white matter hyperintensities (Jokinen et al., 2007), and CC volume loss over a 3-year period predicted longitudinal slowing of CPS in older adults with age-related white matter hyperintensities (Jokinen et al., 2012). The current study provides evidence for the relationship of whole CC volume and CPS over time in a sample of typically aging older adults, providing support for whole CC volume as a proxy of white matter health that predicts change over time.

The positive effects of physical activity on cognitive functioning in healthy older adults, including CPS, have been well-documented (Barnes et al., 2003; Kelly et al., 2014). This study extends these findings, providing evidence that physical activity is associated with robust and stable CPS over time. Although we are not the first to show the beneficial effects of physical activity (Lo et al., 2014; Nouchi et al., 2014), these results offer preliminary evidence that physical activity plays a particularly significant role in the maintenance of CPS over time.

Our finding that two AD risk genes were disproportionally represented in our resilient-agers was surprising. This may be due in part to the small number of subjects meeting criteria as a resilient-ager. Given the roles that *APOE* e4 and *CR1* AA/AG alleles have been reported to play in the deposition of β-amyloid, the breakdown of myelin and cognitive decline (Bartzokis et al., 2007; Lambert et al., 2009; Chibnik et al., 2011), the preponderance of these risk alleles may further suggest the presence of a survival effect among this cohort. While each of these genetic variants confer risk, neither is fully penetrant in its risk effect, and may indicate that individuals in the resilient-ager cohort were never at risk. Furthermore, recent studies investigating the relationship between *CR1* and *APOE* found no evidence for an interaction effect conferring additional risk for AD (Carrasquillo et al., 2011). Thambisetty et al. (2013) recently reported that among non-demented older adults, similar amyloid burden was observed among *APOE* e4 carriers and non-carriers, who were also carriers of *CR1* AA/AG alleles. The relatively high co-occurrence of *APOE* e4 carriers and *CR1* AA/AG carriers in the resilient-ager group stands in accord with these results. On the other hand, these results may suggest the presence of protective factors within the resilient-ager cohort, mitigating the effects of cognitive decline associated with these alleles.

One such potential factor is completed years of education. Kaup et al. (2015) found that among community-dwelling older black and white *APOE* e4 carriers, higher educational level predicted cognitive resilience. While each of the three groups in this study had similar years of completed education, the resilient-ager group reported the highest (mean = 18.4). Physical activity is another factor that has been studied in healthy older adult *APOE* e4 carriers. Smith et al. (2016) recently reported that *APOE* e4 carriers reporting high amounts of leisure time physical activity demonstrated greater white matter integrity (lower fractional anisotropy and greater radial diffusivity) than *APOE* e4 carriers reporting low physical activity. These results suggest that physical activity among *APOE* e4 carriers may offer unique protective effects against white matter demyelination, which in turn could contribute to the maintenance of CPS. While the finding in this study needs replication, it opens exciting lines of inquiry to further examine whether the factors identified in this study or other factors may modify the effects of these genetic risk factors, providing protective effects in the presence of genetic predisposition toward neuropathology.

Results from this study underscore the heterogeneity of cognitive aging even among typically aging older adults. Recent advances in biological aging models posit a multi-determined, mechanistic account of biological aging, with the causal factors of environment, lifestyle factors, genetics, and disease interacting with redox balance, inflammatory status, and vascular health to affect aging, cognitive function, and neurodegeneration (DeCarlo et al., 2014). While our study design does not provide for the analysis or conclusion of the effects of specific variables on others, within such a model, physical activity (PASE) and genotype (*APOE*, *CR1*) represent upstream causal mechanisms with downstream effects on vascular health (insulin resistance) and inflammatory measures (IL-6), which in turn have downstream effects on neurodegeneration (CC volume) and cognitive function (CPS). A strength of the current study is the robust measure of CPS, incorporating 14 task conditions. This method represents a more valid measure of CPS than most studies investigating CPS, which make use of a single measure of CPS (Sheppard and Vernon, 2008). The composite CPS measure used in this study may be particularly well-suited for studies investigating the role of physical exercise or inflammatory processes in older adults.

We acknowledge several limitations of this study. The sample size was relatively small, particularly the cohort of resilient-agers. While by definition this group will likely be smaller, future studies should seek to identify a larger number of these resilient-agers. The majority of our findings dissociated resilient-agers and sub-agers, and these results may reflect differences between normal and abnormal cognitive decline. On the other hand, results of the CC volume, IL-6, and insulin data demonstrated numeric differences and medium effect sizes between the resilient-ager and average-ager groups, so the lack of statistical significance may be an issue of power. We did not incorporate multimodal imaging such as amyloid and tau imaging, or DTI, which would have provided more robust measures of white matter integrity. Our measures of cardiovascular health and inflammation were also limited and the overall impact of these factors on CPS may be even greater. The secondary regression analysis included a reduced sample, which could affect the stability of the model. This initial study included measurement of CPS over time, but we did not investigate longitudinal change in other variables of interest. While the findings in this small group of resilient-agers offers validity to the characterization of unusually successful domain-specific cognitive aging, future studies including more time points and investigating additional cognitive domains will allow for distinct longitudinal aging trajectories to be plotted and more robustly characterized.

The current study adds to a growing body of literature identifying subgroups of older adults that demonstrate uniquely strong domain-specific cognitive abilities that resist age-related cognitive decline. “Super-agers” have previously been characterized in individuals over age 80 with episodic memory performance at least as good as normative values for 50- to 65-year-olds (Rogalski et al., 2013; Gefen et al., 2014, 2015; Sun et al., 2016). This is the first study to our knowledge characterizing older adults who resist age-related change in domains other than episodic memory. While CPS represents a circumscribed cognitive domain, domain-specific cognitive aging has been reported to predict functional decline better than global cognition in elderly populations (Johnson et al., 2007). CPS also plays a mediating role across other cognitive domains including memory and fluid reasoning (Salthouse, 1996). Moreover, slower CPS is associated with functional decline, and serves as a unique early predictor of neurodegenerative processes such as AD (Triebel et al., 2014). Studies investigating unusually successful domain-specific cognitive aging provide a unique opportunity to identify factors that promote healthy cognitive aging trajectories, which may lead to novel interventions for the prevention of age-related cognitive decline.

## Author Contributions

NB made a substantial contribution to the design of the work, the acquisition and interpretation of the work, drafted the work, approved the final version to be published and agreed to be accountable for all aspects of the work. BB, JK made a substantial contribution to the acquisition and interpretation of the work, revised the work critically for important intellectual content, approved the final version to be published and agreed to be accountable for all aspects of the work. JY, DF, and KY made a substantial contribution to the interpretation of the work, revised the work for important intellectual content, approved the final version to be published and agreed to be accountable for all aspects of the work. MW, AK made a substantial contribution to the acquisition of the work, approved the final version to be published and agreed to be accountable for all aspects of the work.

## Conflict of Interest Statement

The authors declare that the research was conducted in the absence of any commercial or financial relationships that could be construed as a potential conflict of interest.

The reviewer ER and handling Editor declared their shared affiliation, and the handling Editor states that the process nevertheless met the standards of a fair and objective review.

## Footnotes

1 http://www.pstnet.com/eprime.cfm

2 http://surfer.nmr.mgh.harvard.edu
