# Supplementary material for: Youthful Processing Speed in Older Adults: Genetic, Biological, and Behavioral Predictors of Cognitive Processing Speed Trajectories in Aging
Source: Front Aging Neurosci. 2017 Mar 10;9:55. doi: 10.3389/fnagi.2017.00055 (PMC5344896; doi:10.3389/fnagi.2017.00055)
Supplement: Supplementary file 1 [file Data_Sheet_1.DOCX]

**SUPPLEMENTAL MATERIAL**

**Cognitive Processing Speed Tasks.**

*Abstract matching 1.* Participants were presented with three arrays of shapes: two choice arrays – a match and a foil – positioned below a third, sample array. They had to decide which of the two choice arrays was most similar to the sample array, then press the corresponding button (left or right). The arrays varied on three dimensions: shape, number, and color. The match always had one more dimension in common with the sample array relative to the foil.

*Distance judgment.* Participants were shown a white circle located approximately in the center of the display, and two additional circles (blue and red) located on the left and right hand- sides of the screen at least one inch away from the center point of an imaginary vertical line that bisects the white center dot. The participant had to judge which of the two additional circles was closest to the white central circle, and push the corresponding key (left or right). The ratio of the distances of the further to the closer dot ranged from 4:1 (easy) to 6:5 (hard).

*Length judgment.* Participants were shown two vertical parallel lines of different lengths, then asked to determine which line was longer and press the corresponding button (left or right). The two lines were displayed such that neither the tops nor bottoms were aligned, thereby requiring participants to make a judgment about actual line length rather than a simple height judgment. For this task, two conditions were equally presented: (1) lines differed in length by 20%, and (2) lines differed in length by 10%.

*Mental rotation.* Participants were shown a letter from the Georgian Cyrillic alphabet that appeared twice on the screen, on the left in its proper (upright) position and on the right in a rotated position; in half of the trials, the right, rotated letter was a mirror- image of the letter on the left so that it did not match the left letter. Participants had to determine whether, after rotation, the two letters were identical (as opposed to being mirror images), and then choose the corresponding key (yes or no). For this task, two conditions were equally presented: (1) the letter on the right would need to be rotated 60˚ to be upright, and (2) the letter on the right would need to be rotated 120˚ to be upright.

*Visual search.* Participants were shown arrays of blue circles and green squares. Subjects had to determine whether a target (a green circle) appeared on the screen and then press the corresponding button (yes or no). Four conditions were equally presented: (1) an array of 16 objects without a target, (2) an array of 16 objects with a target, (3) an array of 24 objects without a target, and (4) an array of 24 objects with a target.

*Shape judgment.* Participants were shown a single shape on top and two additional shapes below. They had to judge which of the two lower shapes (left or right) was most similar to the shape on top and then press the corresponding button (left or right). Each shape was shown in a different color, but color was not relevant to the choice.

*Abstract matching 2.* This task was similar to Abstract Matching 1, except that four dimensions were used instead of three: shape, number, color, and orientation. For this task, three conditions were equally presented: (1) match and sample equivalent on three dimensions, (2) match and sample equivalent on two dimensions, and (3) match and sample equivalent on only one dimension.
